# Acid Treatment Enhances the Antioxidant Activity of Enzymatically Synthesized Phenolic Polymers

**DOI:** 10.3390/polym12112544

**Published:** 2020-10-30

**Authors:** Maria Laura Alfieri, Federica Moccia, Gerardino D’Errico, Lucia Panzella, Marco d’Ischia, Alessandra Napolitano

**Affiliations:** Department of Chemical Sciences, University of Naples “Federico II”, Via Cintia 4, I-80126 Naples, Italy; marialaura.alfieri@unina.it (M.L.A.); federica.moccia@unina.it (F.M.); gderrico@unina.it (G.D.); dischia@unina.it (M.d.); alesnapo@unina.it (A.N.)

**Keywords:** phenolic polymers, biomimetic oxidation, enzymatic synthesis, acid treatment, antioxidant, electron paramagnetic resonance (EPR)

## Abstract

Phenolic polymers produced by enzymatic oxidation under biomimetic and eco-friendly reaction conditions are usually endowed with potent antioxidant properties. These properties, coupled with the higher biocompatibility, stability and processability compared to low-molecular weight phenolic compounds, open important perspectives for various applications. Herein, we report the marked boosting effect of acid treatment on the antioxidant properties of a series of polymers obtained by peroxidase-catalyzed oxidation of natural phenolic compounds. Both 2,2-diphenyl-1-picrylhydrazyl (DPPH) and ferric reducing/antioxidant power (FRAP) assays indicated a remarkable increase in the antioxidant properties for most phenolic polymers further to the acid treatment. In particular, up to a ca. 60% decrease in the EC_50_ value in the DPPH assay and a 5-fold increase in the Trolox equivalents were observed. Nitric oxide- and superoxide-scavenging assays also indicated highly specific boosting effects of the acid treatment. Spectroscopic evidence suggested, in most cases, that the occurrence of structural modifications induced by the acid treatment led to more extended π-electron-conjugated species endowed with more efficient electron transfer properties. These results open new perspectives toward the design of new bioinspired antioxidants for application in food, biomedicine and material sciences.

## 1. Introduction

During the past decade, considerable attention has been directed to implement synthetic mimics of natural phenolic compounds for a broad range of applications, e.g., as active components in resins [1,2,3,4], hydrogels [5,6], adhesives [7,8], nanocarriers [9,10], polymers for packaging [11,12,13,14,15,16], biointerfaces [17,18] and biomaterials [19,20,21,22,23].

Polymers of natural phenolic compounds, in particular, have attracted remarkable interest in view of their lower volatility (with reduced adverse effects), greater stability, and hence higher processability, and lower tendency to be released into the contact medium compared to the corresponding monomers [24,25,26,27,28,29]. Moreover, they are usually endowed with a range of antioxidant properties which further add to the potential of this material [30]. Remarkable examples include, among others, polymerized flavonoids [31,32], lignin-inspired polymers [33], sinapic acid-derived polymers [34], catechol and gallol-type polymers [35].

Among polymerization strategies, enzyme-based processes are emerging as a valid alternative to the use of chemical oxidants, leading to biocompatible and stable, functional materials through environmentally sustainable reactions involving the use of catalytic amounts of enzymes (e.g., laccase, peroxidase, tyrosinase or polyphenol oxidase [36,37,38]) and easily available low-cost oxidants such as hydrogen peroxide.

In particular, peroxidase enzymes are able to catalyze, in a highly efficient manner, the oxidative coupling of phenols and substituted phenols under mild and eco-friendly reaction conditions. The reaction on monophenolic substrates proceeds via one-electron oxidation leading to the generation of phenoxyl radical intermediates, which evolve via sequential oxidative coupling steps through C-C or C-O-C bonding [39,40]. In the case of di- and tri-phenolic compounds, semiquinone radicals are formed which can dimerize or undergo disproportionation, with the generation of quinonoid species that can couple with reduced phenolic species through ionic mechanisms [41,42,43]. Relevant examples of phenolic polymers prepared by peroxidase-catalyzed reactions include poly(caffeic acid methyl ester) (PolyCAME), polypyrogallol and polyguaiacol as sustainable stabilizers toward thermal and photo-oxidative degradation of polyethylene or polypropylene [44,45,46,47], polytyrosol, with potential application in tissue engineering due to its antioxidant and osteogenic activity [48], polymers from 4-hydroxyphenylacetic acid, hydroxytyrosol and chlorogenic acid for application in sensing [49] and polymeric flavonoids [50].

Recently, a systematic investigation of the antioxidant properties coupled with a detailed analysis of the electron paramagnetic resonance (EPR) features of nine biocatalytically produced phenolic polymers has been reported [51]. Moreover, the possibility to improve the electron transfer properties of bioinspired polymers from caffeic and ferulic acid by an acid treatment previously developed for phenol-rich agri-food wastes has been recently investigated [52].

Based on these findings, we report herein the effects of the acid treatment on the antioxidant and radical-scavenging properties of nine polymers prepared by horseradish peroxidase (HRP)-H_2_O_2_-catalyzed oxidation of easily accessible natural phenolic compounds. Spectroscopic analysis was also performed with the aim to obtain information on the structural modifications induced by the acid treatment.

## 2. Materials and Methods

### 2.1. General Experimental Methods

#### 2.1.1. Materials

Horseradish peroxidase (HRP), 30% hydrogen peroxide, 2,2-diphenyl-1-picrylhydrazyl (DPPH), iron (III) chloride (97%), 2,4,6-tris(2-pirydyl)-s-triazine (TPTZ) (≥98%), (±)-6-hydroxy-2,5,7,8-tetramethylchromane-2-carboxylic acid (Trolox) (97%), ethylenediaminetetraacetic acid (EDTA) (>99%), nitroblue tetrazolium (NBT) chloride (98%), sodium nitroprusside (SNP), N-(1-naphthyl)ethylenediamine dihydrochloride, sulphanilamide, gallic acid (≥97.5%), pyrogallol, catechol, 4-methylcatechol, caffeic acid, ferulic acid, *p*-coumaric acid, vanillic acid, tyrosol and quercetin were obtained from Sigma-Aldrich (Milan, Italy).

#### 2.1.2. Characterization

UV–Vis spectra were recorded using a Hewlett Packard 8453 Agilent spectrophotometer. Electron paramagnetic resonance (EPR) measurements were performed using a Bruker Elexys E-500 spectrometer equipped with a superhigh sensitivity probe head. The samples were transferred to flame-sealed glass capillaries, which in turn were coaxially inserted in a standard 4 mm quartz sample tube. Measurements were performed at room temperature. The instrumental settings were as follows: sweep width, 100 G; resolution, 1024 points; modulation amplitude, 1.0 G; scansion time 20.97 s. The amplitude of the field modulation was preventively checked to be low enough to avoid detectable signal overmodulation. Number of scans and microwave power were optimized to avoid microwave saturation of the resonance absorption curve. For power saturation experiments, the microwave power was gradually incremented from 0.02 to 164 mW. The *g* value and the spin density were evaluated by means of an internal standard, Mn^2+^-doped MgO, prepared by a synthetic protocol reported in the literature [53]. Since sample hydration was not controlled during the measurements, spin density values have to be considered as order of magnitude estimates.

Attenuated total reflectance (ATR)-FTIR spectra were recorded on a Nicolet 5700 Thermo Fisher Scientific instrument.

^1^H NMR spectra were recorded in DMSO-d_6_ at 400 or 500 MHz on Bruker or Varian instruments.

### 2.2. Preparation of the Phenolic Polymers

The proper phenol (200–500 mg) was solubilized in ethanol and added to 0.1 mol/L phosphate buffer at pH 6.8 (10 mmol/L phenol final concentration) containing 1% KCl (ethanol/buffer ratio = 1:4 *v*/*v*) [51]. HRP (final concentration 2 U/mL) and 30% H_2_O_2_ (final concentration 20 mmol/L) were then added in two aliquots, within 1 h of each other. The mixture was kept under magnetic stirring overnight, acidified with 3 mol/L HCl up to pH 3 and kept at 4 °C for 24 h. The precipitate was then recovered by centrifugation (8836× *g*, 4 °C, 30 min), washed with 0.1 mol/L HCl and freeze-dried.

### 2.3. Acid Treatment of the Phenolic Polymers

Each polymer (100 mg) was treated with 7 mL of 6 mol/L HCl under stirring at 100 °C for 24 h. After cooling at room temperature, the mixture was centrifuged (4123× *g*, 4 °C, 15 min) and the precipitate was washed three times with water and recovered by freeze drying.

### 2.4. DPPH Assay

A 0.33 mg/mL polymer solution in DMSO (30–360 µL) was added to 2 mL of a freshly prepared 0.2 mmol/L solution of DPPH in ethanol and the mixture was taken under vigorous stirring at room temperature [51,54]. After 10 min, the absorbance at 515 nm was measured. Trolox was used as the reference antioxidant. Experiments were run in triplicate.

### 2.5. FRAP Assay

A 0.33 mg/mL polymer solution in DMSO (5–500 µL) was added to 3.6 mL of a freshly prepared solution containing 1.7 mmol/L FeCl_3_ and 0.83 mmol/L 2,4,6-tris(2-pirydyl)-s-triazine in 0.3 mol/L acetate buffer (pH 3.6) [51,55]. The mixture was taken under vigorous stirring at room temperature and monitored at 593 nm after 10 min. Results were expressed as Trolox equivalents (eqs). Experiments were run in triplicate.

### 2.6. Nitric Oxide (NO)-Scavenging Assay

A 0.33 mg/mL polymer solution in DMSO (600 µL) was added to 6 mL of freshly prepared 10 mmol/L solution of SNP in 0.2 mol/L phosphate buffer (pH 7.4), and the mixture was taken under vigorous stirring at room temperature [51,56]. After 2 h, 1 mL of the mixture was added to 2 mL of Griess reagent (0.5% sulfanilamide and 0.05% *N*-(1-naphthyl)ethylenediamine dihydrochloride in 2.5% phosphoric acid), and the absorbance at 540 nm was measured. Results were expressed as percentage of reduction in the absorbance at 540 nm of a control mixture run in the absence of a sample. Quercetin was used as the reference antioxidant. Experiments were run in triplicate.

### 2.7. Superoxide-Scavenging Assay

A 0.33 mg/mL polymer solution in DMSO (100 µL) was added to 0.05 mol/L ammonium hydrogen carbonate buffer (pH 9.3) containing 0.33 mmol/L EDTA, 0.01 mmol/L NBT and 3.3 mmol/L pyrogallol [51,57]. The mixture was taken under vigorous stirring, and after 5 min, the absorbance at 596 nm was measured. Results were expressed as percentage of reduction in the absorbance at 596 nm of a control mixture run in the absence of a sample. Quercetin was used as the reference antioxidant. Experiments were run in triplicate.

## 3. Results and Discussion

### 3.1. Effects of Acid Treatment on the Antioxidant and Radical-Scavenging Properties of the Enzymatically Synthesized Phenolic Polymers

#### 3.1.1. Preparation of the Acid-Treated Phenolic Polymers

**Figure 1:**
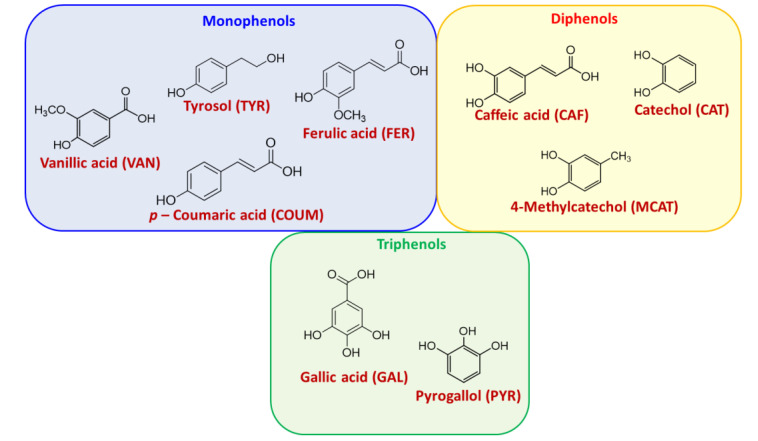
Natural phenols used for the preparation of polymers.

The phenolic polymers were prepared using HRP and H_2_O_2_ in phosphate buffer (pH 6.8) as previously described [51], starting from three distinct sets of monomers, that is, monophenols, diphenols and triphenols (Figure 1). The phenolic polymers were easily recovered by precipitation favored by the presence of KCl in the reaction medium, and then subjected to the acid treatment under experimental conditions optimized in a previous work, underlining the importance of a high temperature and HCl concentration to achieve a significant improvement of the antioxidant properties [58].

In particular, the polymers were exposed to 6 mol/L HCl at 100 °C for 24 h and recovered by centrifugation in variable yields as shown in Table 1, suggesting the occurrence in some cases of a significant breakdown of the polymer backbone, resulting in a change of the solubility properties and hence low isolation yields of the materials separated from the acid solution.

#### 3.1.2. DPPH and FRAP Assays

**Figure 2:**
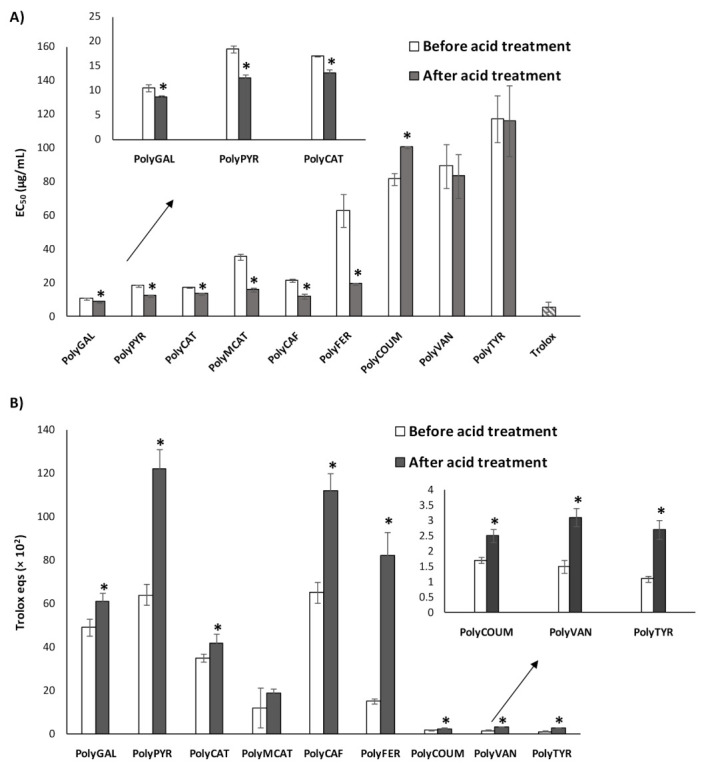
Antioxidant properties of the phenolic polymers before [51] and after acid treatment. (**A**) DPPH and (**B**) FRAP assays. Reported are the mean ± SD values of at least three experiments. Values marked with asterisks are significantly different from those of the corresponding polymer before acid treatment (*p* < 0.05, Microsoft Excel Student’s *t*-test).

The antioxidant properties of the acid-treated phenolic polymers were first evaluated by two widely used chemical assays, i.e., the DPPH and FRAP assays. The results are reported in Table 2 and Figure 2, in comparison with those obtained for the untreated samples [51]. The results of the DPPH assay are expressed as EC_50_, that is, the concentration at which a 50% DPPH reduction is observed. Results of the FRAP assay are expressed as equivalents of the reference antioxidant Trolox. Generally, as expected, PolyGAL and PolyPYR deriving from trihydroxyphenols exhibited the highest antioxidant capability, which was comparable or in some cases even higher than that of Trolox. Diphenolic-based polymers also displayed low EC_50_ values in the DPPH assay and relatively high Trolox eqs, as particularly evident in the case of PolyCAF and PolyCAT. On the contrary, acid-treated polymers from monophenolic compounds showed significantly lower reducing power with the exception of PolyFER that showed EC_50_ and Trolox eqs values in the same order of magnitude as polymers from tri- and diphenolic compounds.

When compared with the starting polymers (Figure 2), the activating effect of the acid treatment was evident in the case of the tri- and diphenol-derived polymers, with a lowering of the EC_50_ values in the DPPH assays ranging from 1.3- for PolyCAT to 2.3-fold in the case of PolyMCAT. On the contrary, no or a low effect of the acid treatment was observed for the polymers prepared from monophenols, with the only exception of PolyFER. As already reported [52], the remarkable activation of the antioxidant properties of this polymer can be attributed to the occurrence of structural modifications resulting in more extended π-electron-conjugated species and in a higher number of free phenolic functions. The occurrence of the demethylation process as a consequence of the acid treatment was ruled out based not only on the previously reported spectroscopic evidence [52], but also considering that no improvement of the antioxidant properties was observed in the case of PolyVAN. Notably, a slight worsening of the DPPH-reducing properties was detected for PolyCOUM.

The activating effect of the acid treatment on the antioxidant properties of the phenolic polymers was found to be less specific in the case of the FRAP assay (Figure 2B), since an improvement of the reducing power was observed for all the materials tested to a variable extent. Noteworthy is the roughly doubling or even sextupling of the Trolox equivalents observed for PolyPYR, PolyCAF and PolyFER.

The differences between the results obtained in the DPPH and FRAP assays can be interpreted based on the different solubilities of the polymers in the aqueous medium used in the FRAP assay compared to the organic solvent in which the DPPH assay was performed, although a good correlation (R^2^ = 0.87) was found between the antioxidant properties exhibited by the acid-treated polymers in the two assays (Figure S1). This would suggest that the same components responsible for the antioxidant properties observed in an organic solvent also determined the iron-reducing properties in the aqueous medium.

#### 3.1.3. NO- and Superoxide-Scavenging Assays

With the aim to assess other properties of biological relevance to broaden the application fields of the acid-treated phenolic polymers, in a further series of experiments, the NO- and superoxide-scavenging capacities of the different samples were evaluated and compared with those previously reported for the untreated polymers [51] (Table 3 and Figure S2).

The results of the NO-scavenging assay suggested again a higher trapping efficiency of the triphenolic-based polymers PolyGAL and PolyPYR, followed by PolyCAT, with all three being more active than the reference compound quercetin. A good activity was also found in the case of PolyCAF and PolyMCAT, whereas monophenolic-based polymers showed NO-trapping capabilities lower than 20%.

A similar trend was observed in the superoxide-scavenging assay, although in this case, PolyFER showed trapping properties comparable to those observed with tri- and diphenol-derived polymers.

The comparison between the NO-scavenging properties of acid-treated and untreated samples showed no significant variations between the two series (Figure S2A), except for PolyCAF which was found to be 5-fold more active after the acid treatment. Halving of the NO-scavenging ability was instead observed in the case of PolyCOUM, whereas PolyTYR completely lost its already poor effectiveness.

The same applied to the superoxide assay, with an even more marked decrease (*ca.* 70%) in the scavenging properties of PolyCOUM. Notably, PolyMCAT and PolyVAN showed a 4- and 20-fold improvement of the activity after the acid treatment, respectively (Figure S2B).

The variability in the activating effects induced by the acid treatment on the radical-scavenging properties of the phenolic polymers can be likely ascribed to the variegated and complex mechanisms operating in these assays, in agreement with what has previously been reported [51].

### 3.2. Spectroscopic Insights into the Structural Modifications Induced by the Acid Treatment on the Enzymatically Synthesized Phenolic Polymers

#### 3.2.1. UV–Vis Analysis

**Figure 3:**
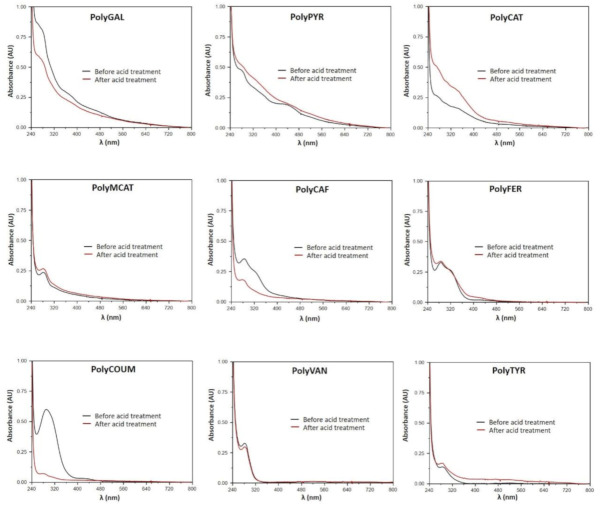
UV–vis spectra of 0.01 mg/mL methanolic solutions (from dilution of a 0.33 mg/mL solution in DMSO) of the enzymatically synthesized phenolic polymers before (**black line**) and after (**red line**) the acid treatment.

To gain insights into the chemical modifications induced by the acid treatment on the HRP/H_2_O_2_-synthesized phenolic polymers, DMSO solutions of the different samples were analyzed by UV–vis spectroscopy after proper dilution in methanol. As shown in Figure 3, no changes were observed in the spectra of the monophenol-derived polymers PolyTYR and PolyVAN, whereas an almost total abatement of the absorption at 290 nm was evident in the case of PolyCOUM, indicating the occurrence of extensive structural modifications of the polymer backbone. As previously reported [52], the other hydroxycinnamic acid-derived samples, that is, PolyCAF and PolyFER, also underwent significant structural transformations further to the acid treatment. As far as the other polymers are concerned, the triphenol-derived PolyGAL and PolyPYR showed a general loss of the spectroscopic features, whereas an increase in the absorbance with no significant modifications in the spectrum characteristics was observed for PolyCAT. On the contrary, no apparent changes in the UV–vis absorption properties were detected for PolyMCAT.

All these results are reasonably in line with those of the antioxidant assays, showing a more or less marked abatement of the reducing and radical-scavenging properties for PolyCOUM, likely as a consequence of detrimental extensive structural modifications induced by the acid treatment, reflected also in the low recovery yields reported in Table 1. On the other hand, the broadening of the UV–vis spectrum observed for PolyGAL and PolyPYR and, in part, for PolyCAT would be indicative of the occurrence of chemical reactions resulting in the formation of more varied species or structural units, possibly characterized by a more extended π-electron conjugation and hence endowed with higher antioxidant properties, in agreement with what has recently been reported for PolyCAF and PolyFER [52].

#### 3.2.2. EPR Analysis

**Figure 4:**
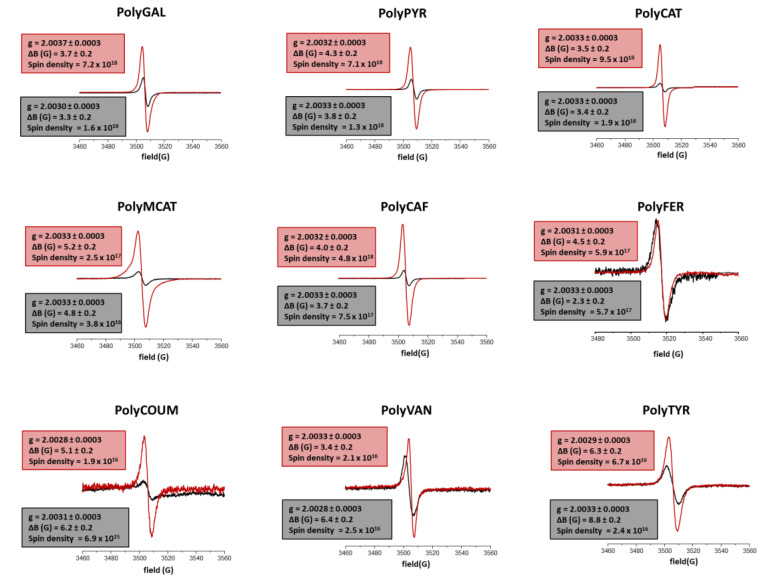
Solid-state EPR spectra of the enzymatically synthesized phenolic polymer before (**black traces**) and after (**red traces**) acid treatment.

EPR spectroscopy has been recently proven to be a promising approach to inquire into the structural basis of the antioxidant activity of phenolic polymers, these being characterized by the presence of intrinsic free radical centers [51,52]. Table 4 collects the EPR parameters determined for the nine enzymatically synthesized phenolic polymers subjected to the acid treatment, whose spectra are shown in Figure 4 together with those recorded for the untreated samples.

The spectra of all the acid-treated polymers exhibited a *g* value consistent with the presence of carbon-centered radicals [51,59]. In agreement with what has previously been reported for the untreated samples [51], significative differences were evident in the spin density, with the highest values found for triphenolic- and diphenolic-based polymers. Slighter differences were found in the signal amplitude (ΔB) values, although an increasing trend was apparent moving from trihydroxy- (4.0 ± 0.3) to dihydroxy- (4.2 ± 1.2) and monohydroxy-derived (4.8 ± 1.5) polymers, in agreement again with previous data [51].

The comparison between the EPR parameters of treated and untreated samples (Figure 4 and Table S1) showed an increase in the spin density values ranging from 4.5- to 6.6-fold for the triphenol- and diphenol-derived acid-treated polymers, while only ca. 2.8 higher values were determined after the acid treatment for the monophenolic polymers PolyCOUM and PolyTYR. Notably, no substantial variations compared to the starting samples were observed for methoxyhydroxy-derived PolyVAN and PolyFER. As far as the ∆B values are concerned, less marked differences were observed before and after the acid treatment, with the exception, in this case, of PolyFER, for which a doubling was observed. On the contrary, acid-treated monophenol-based polymers PolyCOUM, PolyVAN and PolyTyr showed a lower ∆B in comparison with untreated materials.

Based on all these data, the acid treatment seemed to significantly affect the structure of the hydroxycinnamic-derived polymers PolyCAF and PolyFER, as well as of those characterized by the presence of highly oxygenated, planar moieties, as is the case of PolyGAL, PolyPYR and PolyCAT. These two classes of polymers could indeed undergo extensive chemical modifications involving, e.g., dehydration and/or oxidation processes driven by the acidic pH and high temperature, as previously demonstrated in the case of PolyCAF and PolyFER [52]. The occurrence of these processes would likely result in the formation of more chemically heterogeneous species accompanied by a loss of specific and well-defined chromophoric features, and hence in a broadening of the UV–vis spectrum, as reported in Figure 3. This would also be in line with the results of the EPR investigation, generally highlighting an increase in the spin density and/or ∆B values further to the acid treatment. Acid-induced dehydration processes leading to polyacetylene-like conjugated polymers have also been reported to increase electron conductivity [60,61]. Indeed, as previously discussed, a higher spin density value would be indicative of structural features able to stabilize unpaired electrons and hence to sustain electron transfer processes, such as highly π-conjugated systems [51]. On the same line, the increase in ∆B values can be taken as a probe of a larger number of π-stacking interactions resulting from a rearrangement of supramolecular aggregation as a consequence of the increase in planar units [52,62]. The possibility that the acid treatment breaks weak C-O linkages inducing depolymerization processes and hence leading to an increase in low-molecular weight molecules exhibiting a higher number of phenolic OH moieties and, as a consequence, a higher spin density cannot be ruled out. All these structural modifications would be, in part, responsible for the observed boosting effects on the antioxidant properties of the phenolic polymers. In support of this hypothesis, an opposite trend was observed for the monophenol-derived polymers PolyCOUM, PolyVAN and PolyTyr, which were the ones that “benefited” least from the acid treatment.

#### 3.2.3. ATR-FTIR Analysis

**Figure 5:**
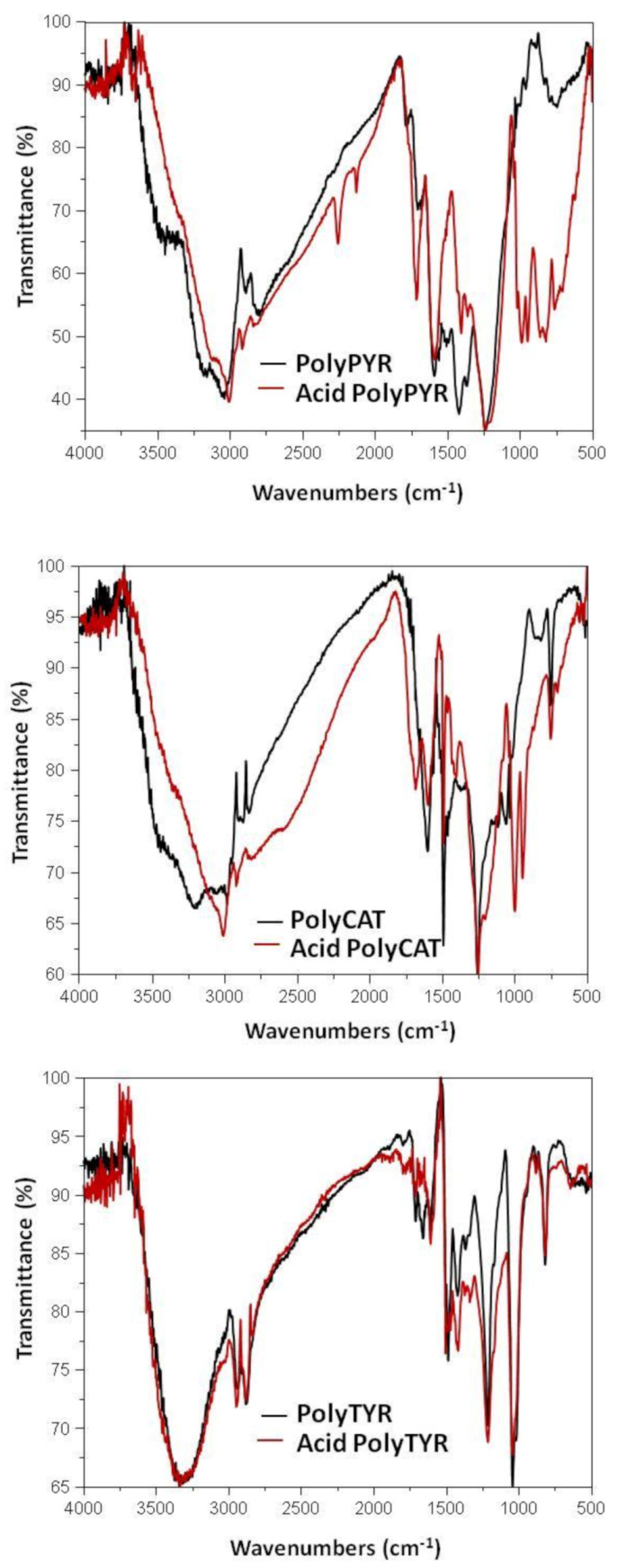
ATR-FTIR spectra of selected enzymatically synthesized phenolic polymers before (**black line**) and after (**red line**) the acid treatment.

To further probe the occurrence of the chemical modifications discussed above during the acid treatment, selected polymers from each class, that is, PolyPYR, PolyCAT and PoyTYR, were analyzed by ATR-FTIR spectroscopy before and after the treatment. The spectra shown in Figure 5 confirmed the occurrence of significant structural modifications further to the acid treatment in the case of PolyPYR and PolyCAT, whereas no marked differences were observed for PolyTYR, in agreement with the results of UV–vis analysis. In particular, the appearance of new bands at 1000–900 cm^−1^ likely due to =C–H bending vibrations was evident for both PolyPYR and PolyCAT, suggesting additional double bond formation, resulting in more extended π-conjugation systems. C=C stretching bands in the region 1670–1600 cm^−1^ were also particularly intense in the spectrum of acid-treated PolyPyR.

#### 3.2.4. ^1^H NMR Analysis

In another series of experiments, the same selected polymers as above, that is, PolyPYR, PolyCAT and PoyTYR before and after the acid treatment, were analyzed also by ^1^H NMR (Figure S3). Again, no significant variation in the spectral features was apparent in the case of PolyTyr, apart from a general loss of resolution in both the low- and high-field region of the spectrum. The same applies to PolyCAT, whereas an almost complete loss of proton signals was observed in the acid-treated PolyPYR sample. This finding would be in agreement with the occurrence of chemical processes leading to novel and more varied structural units, possibly involving the formation of new C-C/C=C bonds.

## 4. Conclusions

The effects of an acid treatment on the antioxidant activities of a series of enzymatically synthesized phenolic polymers have been reported, with a view to further improve the properties and the possible application fields of these promising materials. Spectroscopic analysis indicated the occurrence of significant structural modifications leading in most cases to the generation of more extended π-conjugated systems, responsible for the improvement of the antioxidant properties observed.

The acid treatment also led to selective improvement in the radical-scavenging properties of the phenolic polymers, strongly activating, for example, PolyCAF as a NO scavenger and PolyMCAT and PolyVAN as superoxide trappers.

**Figure 6:**
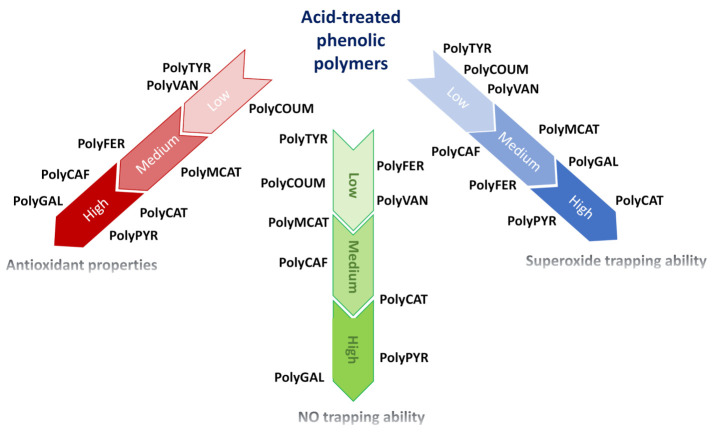
Overview of the antioxidant and radical-scavenging properties of acid-treated enzymatically synthesized phenolic polymers. Position along the arrows indicates the relative efficiency of the polymers.

In conclusion, the remarkable improvement in the reducing and radical-scavenging properties may lead, in some cases, to materials as active as reference antioxidants (Figure 6), opening new perspectives for the full exploitation of these low-cost and environmentally friendly materials. Potential applications include the use as functional additives to stabilize polymers typically used in the food packaging industry against oxidative degradation processes, reducing matrices for the production of metal nanoparticles, antioxidant ingredients for cosmetic products or semiconducting materials. The low cytotoxicity exhibited by related materials subjected to the same activation treatment [63,64], the high accessibility of the starting phenols and of the biocatalytic system needed for their preparation and the cost-effectiveness of the acid activation protocol further contribute to the interest in these materials as a favorable alternative to phenol-containing natural matrices.

## Figures and Tables

**Table 1:** Recovered yields of acid-treated phenolic polymers ^1^.

Sample	Yield (*w*/*w*)
PolyGAL	65%
PolyPYR	100%
PolyCAT	70%
PolyMCAT	67%
PolyCAF	83%
PolyFER	67%
PolyCOUM	29%
PolyVAN	62%
PolyTYR	75%

^1^ Reported are the mean values of three experiments (SD ≤ 5%).

**Table 2:** Antioxidant properties of the acid-treated phenolic polymers ^1^.

Sample	EC_50_ (µg/mL) ^2^ (DPPH Assay)	Trolox eqs (×10^2^) (FRAP Assay)
PolyGAL	8.8 ± 0.3	61 ± 4
PolyPYR	12.5 ± 0.7	122 ± 9
PolyCAT	13.5 ± 0.7	42 ± 4
PolyMCAT	16 ± 1	19 ± 2
PolyCAF	12 ± 1	115 ± 8
PolyFER	19.5 ± 0.7	82 ± 11
PolyCOUM	100.5 ± 0.7	2.5 ± 0.2
PolyVAN	84 ± 13	3.1 ± 0.3
PolyTYR	116 ± 21	2.7 ± 0.3
Trolox	6.0 ± 0.8	100

^1^ Reported are the mean ± SD values of at least three experiments. ^2^ EC_50_ is the concentration at which a 50% DPPH reduction is observed.

**Table 3:** Radical-scavenging properties of the acid-treated phenolic polymers ^1^.

Sample	NO Scavenging (%)	Superoxide Scavenging (%)
PolyGAL	67 ± 3	59 ± 9
PolyPYR	66 ± 2	64 ± 3
PolyCAT	58 ± 2	66 ± 1
PolyMCAT	31 ± 1	53 ± 8
PolyCAF	46 ± 6	40 ± 4
PolyFER	9 ± 4	56 ± 3
PolyCOUM	13 ± 5	18 ± 5
PolyVAN	16 ± 5	25 ± 6
PolyTYR	−	1.0 ± 0.9
Quercetin	44 ± 3	74 ± 4

^1^ Reported are the mean ± SD values of at least three experiments.

**Table 4:** Electron paramagnetic resonance (EPR) parameters of the acid-treated phenolic polymers ^1^.

Sample	*g*-Factor	Spin Density (spin g^−1^)	ΔB (G)
PolyGAL	2.0037	7.2 × 10^18^	3.7
PolyPYR	2.0032	7.1 × 10^18^	4.3
PolyCAT	2.0033	9.5 × 10^18^	3.5
PolyMCAT	2.0033	2.5 × 10^17^	5.2
PolyCAF	2.0032	4.8 × 10^18^	4.0
PolyFER	2.0032	5.9 × 10^17^	4.5
PolyCOUM	2.0028	1.9 × 10^16^	5.1
PolyVAN	2.0033	2.1 × 10^16^	3.4
PolyTYR	2.0029	6.7 × 10^16^	6.3

^1^ Experimental uncertainties are ±0.0003 on *g*-factor, ±10% on spin-density and ±0.2 g on ΔB.

## Supplementary Materials

The following are available online at https://www.mdpi.com/2073-4360/12/11/2544/s1, Figure S1. Correlation between EC_50_ (DPPH assay) and Trolox eqs (FRAP assay) values of the acid-treated polymers, Figure S2. NO- and superoxide-scavenging properties of the enzymatically synthesized phenolic polymers before and after acid treatment, Figure S3. ^1^ H NMR spectra of selected phenolic polymers before and after acid treatment, Table S1. EPR parameters of the phenolic polymers before and after acid treatment.
