# Supplementary material for: Acid Treatment Enhances the Antioxidant Activity of Enzymatically Synthesized Phenolic Polymers
Source: Polymers (Basel). 2020 Oct 30;12(11):2544. doi: 10.3390/polym12112544 (PMC7692195; doi:10.3390/polym12112544)
Supplement: Supplementary file 1 [file polymers-12-02544-s001.pdf]

Acid Treatment Enhances the Antioxidant Activity of Enzymatically Synthesized Phenolic Polymers

Maria Laura Alfieri, Federica Moccia, Gerardino D'Errico, Lucia Panzella *, Marco d'Ischia and Alessandra Napolitano

Department of Chemical Sciences, University of Naples "Federico II", Via Cintia 4, I-80126 Naples, Italy; marialaura.alfieri@unina.it (M.L.A.); federica.moccia@unina.it (F.M.); gderrico@unina.it (G.D.E.); dischia@unina.it (M.d.I.); alesnapo@unina.it (A.N.)

* Correspondence: panzella@unina.it; Tel.: +39-081-674131

Table of contents **Page**

Figure S1. Correlation between EC_{50} (DPPH assay) and Trolox eqs (FRAP assay) values of the acid-treated polymers. 2

Figure S2. NO and superoxide scavenging properties of the enzymatically synthesized phenolic polymers before and after acid treatment. 3

Table S1. EPR parameters of the phenolic polymers before and after acid treatment. 4

Figure S3. ^{1}H NMR spectra of selected phenolic polymers before and after acid treatment. 5

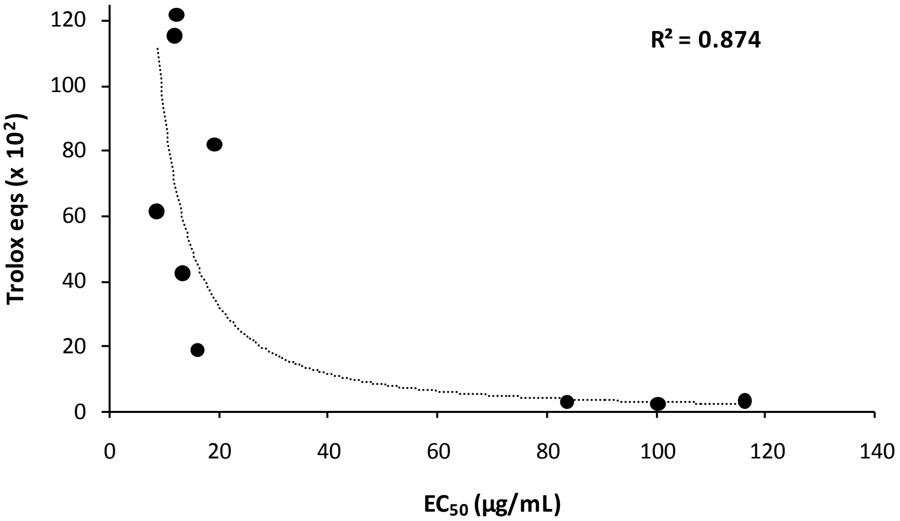

Figure S1. Correlation between EC_{50} (DPPH assay) and Trolox eqs (FRAP assay) values of the acid-treated polymers.

A)

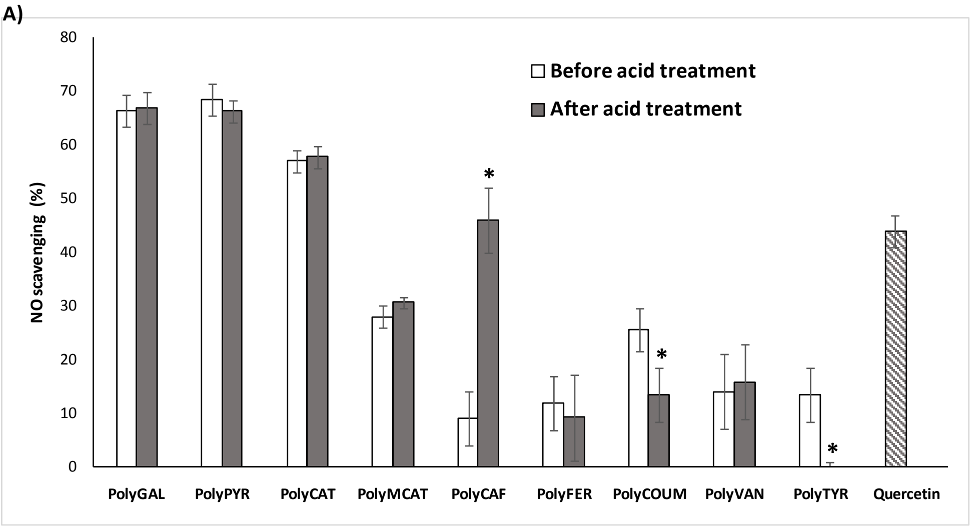

B)

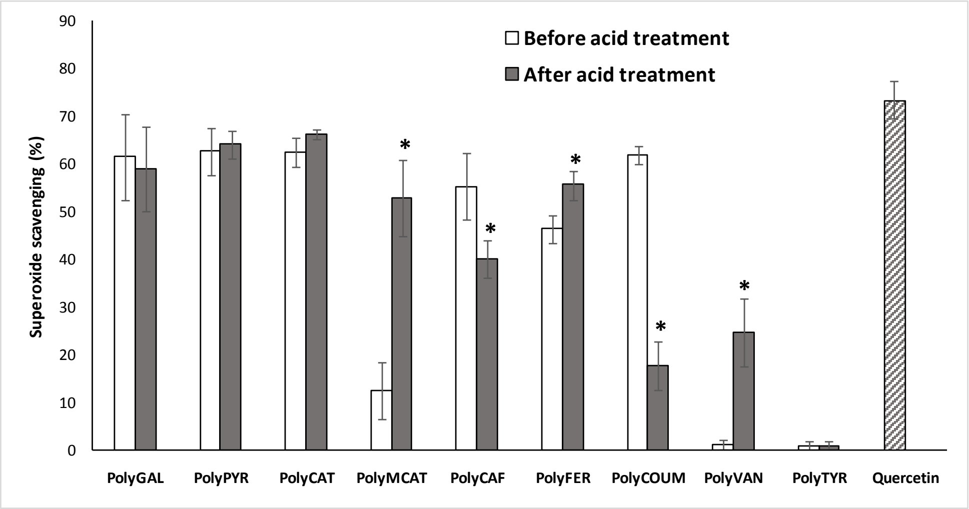

Figure S2. **A**) NO and **B**) superoxide scavenging properties of the phenolic polymers before and after acid treatment. Reported are the mean ± SD values of at least three experiments. Values marked with asterisks are significantly different from those of the corresponding polymer before acid treatment (P<0.05, Microsoft Excel Student's t-test).

Table S1. EPR parameters of the phenolic polymers before and after acid treatment.[1]

Polymer	g-factor	Spin density (spin g^{-1})	ΔB (G)
PolyGAL	2.0030	1.6×10^{18}	3.3
Treated PolyGAL	2.0037	7.2×10^{18}	3.7
PolyPYR	2.0033	1.3×10^{18}	3.8
Treated PolyPYR	2.0032	7.1×10^{18}	4.3
PolyCAT	2.0033	1.9×10^{18}	3.4
Treated PolyCAT	2.0033	9.5×10^{18}	3.5
PolyMCAT	2.0033	3.8×10^{16}	4.8
Treated PolyMCAT	2.0033	2.5×10^{17}	5.2
PolyCAF	2.0033	7.5×10^{17}	3.7
Treated PolyCAF	2.0032	4.8×10^{18}	4.0
PolyFER	2.0033	5.7×10^{17}	2.3
Treated PolyFER	2.0032	5.9×10^{17}	4.5
PolyCOUM	2.0031	6.9×10^{15}	6.2
Treated PolyCOUM	2.0028	1.9×10^{16}	5.1
PolyVAN	2.0028	2.5×10^{16}	6.4
Treated PolyVAN	2.0033	2.1×10^{16}	3.4
PolyTYR	2.0033	2.4×10^{16}	8.8
Treated PolyTYR	2.0029	6.7×10^{16}	6.3

[1] Experimental uncertainties are ± 0.0003 on g-factor, ± 10% on spin-density and ± 0.2 g on ΔB.

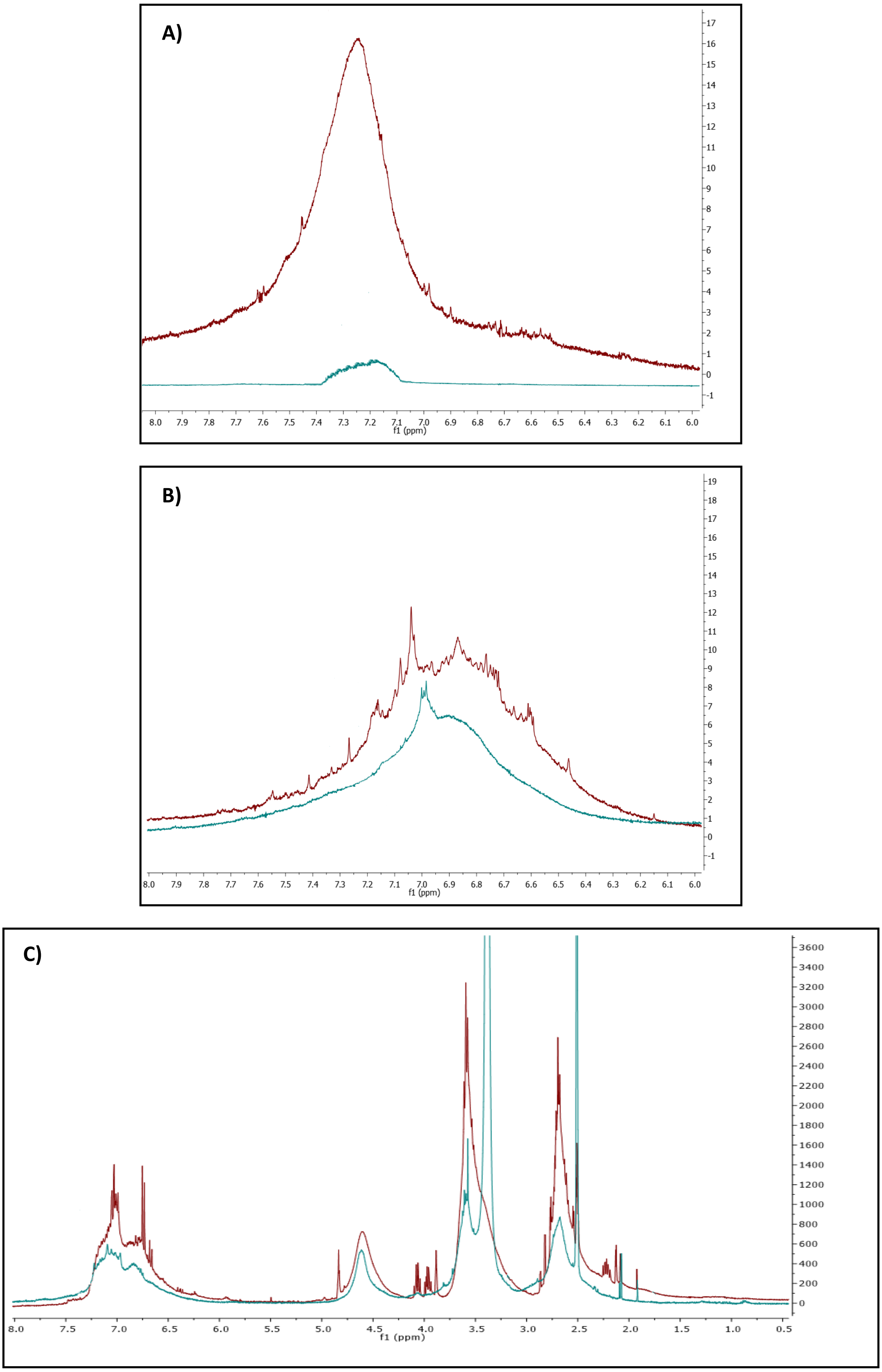

Figure S3. ^{1}H NMR spectra (DMSO-d$_6$) of selected enzymatically synthesized phenolic polymers before (red trace) and after (blue trace) the acid treatment. A) PolyPYR. B) PolyCAT. C) PolyTYR.
